# Ultrastructural Remodeling of the Blood–Brain Barrier and Neurovascular Unit by Lipopolysaccharide-Induced Neuroinflammation

**DOI:** 10.3390/ijms24021640

**Published:** 2023-01-13

**Authors:** Michelle A. Erickson, Tatyana Shulyatnikova, William A. Banks, Melvin R. Hayden

**Affiliations:** 1 Geriatric Research Education and Clinical Center, Veterans Affairs Puget Sound Health Care System, Seattle, WA 98108, USA; 2 Division of Gerontology and Geriatric Medicine, Department of Medicine, University of Washington School of Medicine, Seattle, WA 98104, USA; 3 Department of Pathological Anatomy and Forensic Medicine, Zaporizhzhia State Medical University, Mayakovsky Avenue, 26, 69035 Zaporizhzhia, Ukraine; 4 Department of Internal Medicine, Endocrinology Diabetes and Metabolism, Diabetes and Cardiovascular Disease Center, University of Missouri School of Medicine, One Hospital Drive, Columbia, MO 65211, USA

**Keywords:** blood–brain barrier, brain endothelial cells, caveolae-mediated vesicles, clathrin-mediated vesicles, macropinosomes, microglia, neurovascular unit, receptor-mediated transcytosis, transcytosis, transmission electron microscopy

## Abstract

The blood–brain barrier (BBB) is an interface primarily comprised of brain endothelial cells (BECs), separating the central nervous system (CNS) from the systemic circulation while carefully regulating the transport of molecules and inflammatory cells, and maintaining the required steady-state environment. Inflammation modulates many BBB functions, but the ultrastructural cytoarchitectural changes of the BBB with inflammation are understudied. Inflammation was induced in male 8–10-week-old CD-1 mice with intraperitoneal lipopolysaccharide (LPS), using a regimen (3 mg/kg at 0, 6, and 24 h) that caused robust BBB disruption but had minimal lethality at the study timepoint of 28 h. Perfusion-fixed brains were collected and the frontal cortical layer III regions were analyzed using a transmission electron microscopy (TEM). The LPS-treated mice had pronounced ultrastructural remodeling changes in BECs that included plasma membrane ruffling, increased numbers of extracellular microvesicles, small exosome formation, aberrant BEC mitochondria, increased BEC transcytosis, while tight junctions appeared to be unaltered. Aberrant pericytes were contracted with rounded nuclei and a loss of their elongated cytoplasmic processes. Surveilling microglial cells were attracted to the neurovascular unit (NVU) of BECs, and astrocyte detachment and separation were associated with the formation of a perivascular space and pericapillary edema. The LPS treatment resulted in numerous ultrastructural aberrant remodeling changes to the neurovascular unit’s BECs, microglia, pericytes, and astrocytes. In summary, a disturbance of the NVU morphology is a consequence of LPS treatment.

## 1. Introduction

The vascular blood–brain barrier (BBB) protects and nourishes the brain by providing a dynamic interface between the blood and the brain tissue. The BBB is comprised of brain endothelial cells (BECs) that have a paucity of pinocytic vesicles, an absence of fenestrae, and the unique expression of paracellular tight and adherens junctions (TJ/AJ) [1,2]. BECs are in constant communication with the other cell types of the brain, most notably pericytes and astrocytes, but also microglia, neurons, and mast cells. This complex of cells is termed the neurovascular unit (NVU) and its intercellular communication determines the characteristics of the BBB [3,4].

Inflammation is a major cause of BBB dysfunction. Inflammation-induced BBB dysfunction includes the disruption of the BBB, resulting in leakage of plasma into the brain, and altered functioning of other activities, such as BBB transporters [5,6,7]. Inflammation-induced BBB dysfunction is implicated in the BBB impairments seen in sepsis, HIV encephalopathy, diabetes mellitus, Alzheimer’s disease, Parkinson’s disease, multiple sclerosis, and many other conditions [8].

A classic model for studying inflammation-induced BBB dysfunction is the administration of lipopolysaccharide (LPS) [9,10]. LPS is a lipid (Lipid A) polysaccharide derived from Gram-negative bacterial plasma membranes and results in inflammation and neuroinflammation [11]. Thus, LPS is a byproduct of both the microbiome and infections and is known to be increased by obesity, metabolic syndrome, and diabetes mellitus [12,13,14]. Besides affecting BECs, LPS is also known to alter the other cells of the NVU [8,9,10]. The effects of LPS on the BBB can be direct, with LPS directly binding to BECs, or indirect, with LPS inducing the release of substances from cells other than BECs which then affect the BBB.

The direct effects of LPS first involve peripherally derived LPS binding to blood-borne lipopolysaccharide-binding protein (LBP) that also incorporates a soluble cluster of differentiation 14 (CD14). This LPS/LBP/CD14 cluster binds to toll-like receptor 4 (TLR4) on BECs. TLR4, a transmembrane protein and a member of the toll-like receptor family, belongs to the pattern recognition receptor (PRR) family [11].

The indirect effects of LPS can be mediated through its actions on peripheral cells, including circulating immune cells, which can release cytokines and chemokines that interact with the luminal surface of BECs and alter BBB functions, including BBB permeability to immune cells. Many cytokines and chemokines can cross the BBB to affect NVU functions, and neuroimmune substances released from the abluminal surface of BECs also affect the NVU [8]. In addition, these changes in NVU functions can affect BBB functions. For example, TLR4 signaling through the phosphatidylinositol 3-kinase/protein kinase B (PI3K/AKT) pathway and the mitogen-activated protein kinase (MAPK) pathway activates nuclear factor-kappa B (NF-κB). In turn, NF-κB serves as the signaling mechanism that initiates the chemokine C-C motif ligand 5/regulated on activation, and normal T cell expressed and secreted (CCL5/RANTES) to attract microglial cells to BECs [15,16]. Because blood-borne LPS has not been demonstrated to cross even a disrupted BBB [17], the effects of LPS on NVU functions are presumed to be by such indirect mechanisms.

Perhaps the best-studied aspect of LPS-induced changes in BBB function is that of disruption. Most of the previous studies have focused on tight junction protein expression and function and have used in vitro models of the BBB. Here, we examine the ultrastructural effects of an in vivo LPS dosing regimen known to have effects on BEC and NVU functions, which include BBB disruption [9].

## 2. Results

### 2.1. LPS Treatment Induced Attracted Perivascular Microglia (atMGCs)

**Figure 1:**
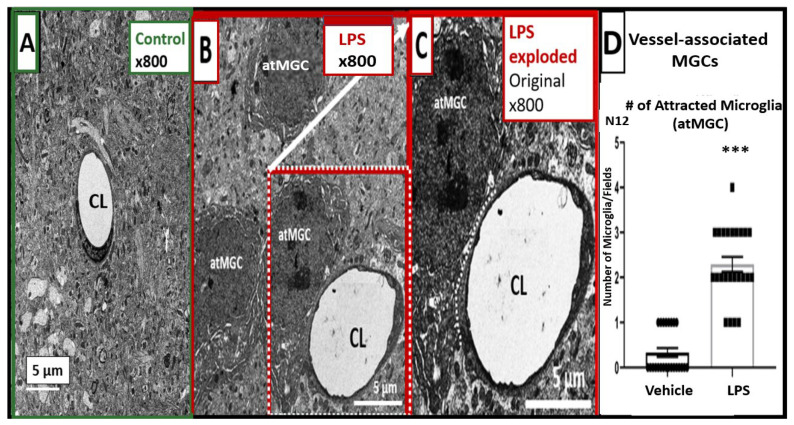
LPS results in increased perivascular-attracted microglial cells (atMGC)s in the cortical layer III of the frontal grey matter in CD-1 male mice. (**A**) demonstrates the neurovascular unit (NVU) of the control group. The perivascular MGCs and atMGCs are rarely seen in the controls. (**B**) depicts the ameboid and attracted MGCs (atMGCs) in the perivascular region of the NVU. Every NVU examined in the LPS-injected mice has at least one perivascular atMGC. (**C**) is an enlarged image from panel (**B**) depicting the close association of the atMGCs (white-dashed line) to BECs comprising the capillary. (**D**) shows the quantification of perivascular atMGC numbers to the NVU; t = 10.21, df = 46, *p* < 0.0001 (***), n = 24 fields from 2 mice per treatment group. Magnifications and scale bars are included. CL = capillary lumen, LPS = lipopolysaccharide.

Microglial recruitment to the brain vasculature occurs with inflammation and is an initial mechanism that can protect against BBB disruption. However, as the inflammatory response progresses, microglia promote BBB disruption [16]. To understand the microglia/BBB interactions in the LPS model used here, we measured the numbers of microglial cells attracted to the brain vasculature (atMGCs), defined as being within 20 μm of a brain vessel, which is consistent with the definitions of vasculature-associated microglia established by other groups [18]. Figure 1 shows the effect of LPS on atMGCs. The results of this ultrastructural study investigating the cortical grey matter in layer III strongly suggest that microglia are attracted to brain capillaries in response to the LPS regimen and adopt an ameboid morphology (Figure 1A–C).

When quantified, the LPS-treated mice had a significant increase in the number of attracted, perivascular MGCs (Figure 1D).

### 2.2. LPS Treatment Induced Increased Numbers of BEC Vesicles, but No Apparent Structural Changes in Tight and Adherens Junctions (TJ/AJ)

**Figure 2:**
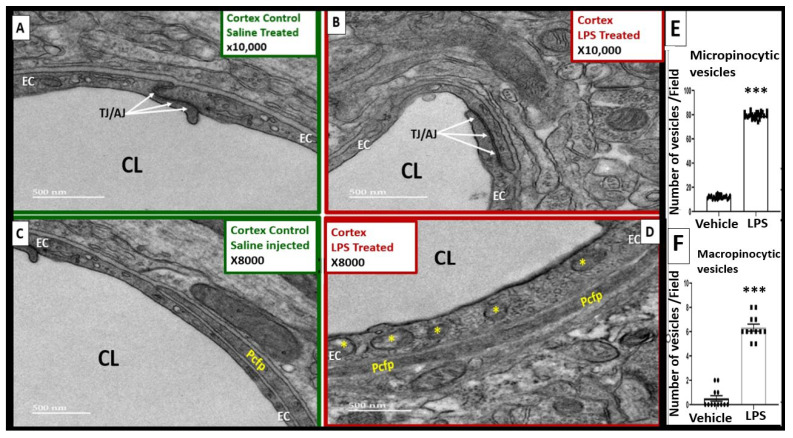
Comparison of numbers of micropinocytic and macropinocytic vesicles in the control and LPS-treated CD-1 mice with intact tight and adherens junctions (TJ/AJ). (**A**,**B**) demonstrate that the TJ/AJs in the LPS-treated mice (**B**) remain intact and similar to the control mice (**A**). (**C**,**D**) depict that macropinocytic vesicles (asterisks) are increased in the LPS-treated models (**D**) compared to the control saline-treated models (**C**), and micropinocytic vesicles are increased in (**D**) compared to the controls in (**A**). (**E**) depicts a significant increase in the number of micropinocytic vesicles (60–70 nm) in the LPS-treated models compared to the control vehicle saline-treated controls: t = 110.1, df = 46, *p* < 0.0001 (***), n = 24 fields from 2 mice per treatment group. (**F**)depicts a significant increase in the number of macropinocytic vesicles (125–300 nm) in the LPS-treated models compared to the saline-treated models: t = 15.95, df = 22, *p* < 0.0001 (***), n = 12 fields from 2 mice per treatment group. CL = capillary lumen; EC = brain endothelial cells; Pcfp = pericyte foot process, LPS = lipopolysaccharide.

The ultrastructural studies depicted an increase in BEC vesicles in the LPS-treated mice. In contrast, TJ/AJs at apposing BEC membranes appeared to be unchanged in these transmission electron microscope (TEM) observational studies (Figure 2A vs. Figure 2B).

**Figure 3:**
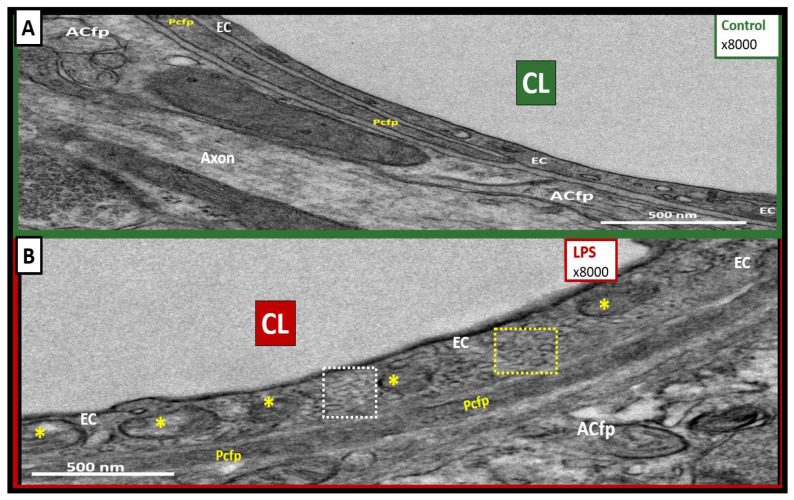
Increased macropinocytosis and micropinocytosis in the lipopolysaccharide (LPS)-treated models. (**A**) demonstrates, in a control mouse brain, an EC with no macropinosomes and a paucity of micropinosomes. (**B**) depicts an LPS-treated mouse brain that clearly depicts an increase in macropinocytic vesicles (yellow asterisks) and micropinocytic vesicles compared to the control model in (**A**) (see bar graphs for statistical differences in Figure 2E,F). Note the apparently uncoated caveolae-mediated micropinocytic vesicles—micropinosomes (white dashed box)—and the apparently coated clathrin-mediated micropinosomes (yellow dashed box) with increased electron-dense coatings. Magnification ×8000; scale bar = 500 nm. ACfp = astrocyte foot processes; CL = capillary lumen; EC = brain endothelial cells; Pcfp = pericyte foot processes, LPS = lipopolysaccharide.

An increase was seen in vesicles ranging in diameter between 125 and 300 nm, which we termed macropinocytic vesicles, or macropinosomes (Figure 2C–F and Figure 3B), and between 60 and 70 nm, which we termed micropinocytic vesicles (Figure 2C–E and Figure 3B). A quantification of macro- and micropinocytic vesicles is shown in Figure 2E,F. An enlarged image that more clearly displays both types of vesicles in the LPS-treated models compared to the saline-treated models is depicted in Figure 3.

Although prior works investigating the effects of inflammation on the ultrastructural changes of the BBB reported vesiculotubular structures, which are patent transendothelial channels formed by the fusion of pinocytic vesicles [19,20], these were not observed in the LPS model used here.

### 2.3. LPS Treatment Induced Pericyte (Pc) Remodeling and Vasogenic Edema

**Figure 4:**
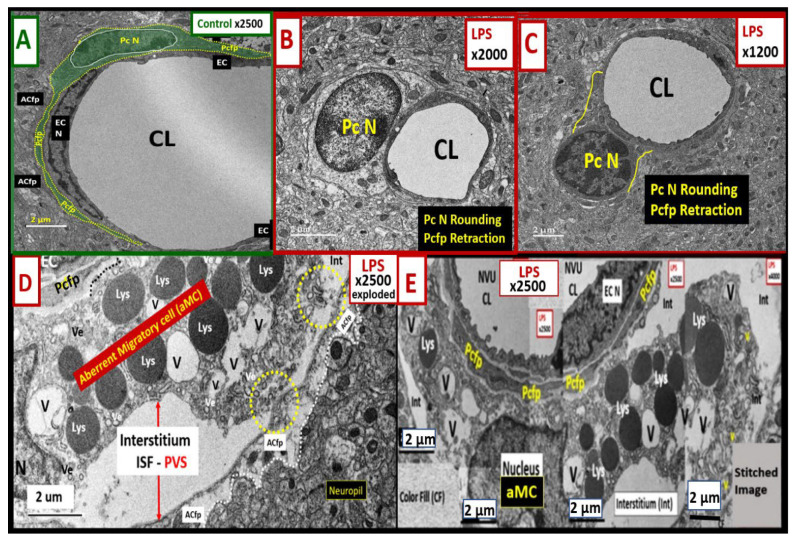
LPS induces pericyte (Pc) remodeling. (**A**) demonstrates a normal control Pc (pseudo-colored green outlined in yellow) ensheathing the neurovascular unit (NVU) brain endothelial cell (EC). Note the elongated Pc nucleus that is similar to the elongated EC and its elongated ensheathing pericyte foot process (Pcfp). (**B**,**C**) depict Pc contraction with Pc nuclei rounding and loss of Pcfp elongation and ensheathment of the BEC. (**D**) depicts an enlarged image of an aberrant migratory cell (aMC) in Microsoft Paint with an intact scale bar within an expanded perivascular space. This image is to demonstrate the lysosomes (Lys), vacuoles (V), and vesicles (Ve) that are typical of a highly synthetic granular pericyte. Note the contacts made between the aMC and the Pcfp (dashed black line) and ACfps (yellow circles with dashed lines), which allow for bidirectional communication. (**E**) depicts multiple images carefully stitched together to better appreciate the abnormal remodeling of this aMC. Note the activated BEC plasma membrane ruffling. Magnification ×2500; scale bars = 2 μm in panels (**A**,**D**,**E**). Magnification ×2000; ×1200; scale bars = 2 μm in panels (**B**,**C**), respectively. ACfp = astrocyte foot process; CL = capillary lumen; EC = brain endothelial cell; EC N = brain endothelial cell nucleus; Pc N = pericyte nucleus; PVS = perivascular space, LPS = lipopolysaccharide.

Pcs are an essential component of NVU, supporting the integrity of the BBB [21]. Here, we found that LPS resulted in Pc remodeling (Figure 4).

Pcs lose their elongated nucleus and perivascular cytoplasmic elongated foot processes, adopting rounded nuclei and hypertrophic changes with a loss of elongated cytoplasmic foot processes (Figure 4B,C), including increased lysosomes, vacuoles, and vesicles (Figure 4D,E). Some regions have marked pericapillary edema with a marked increase in the perivascular space and are associated with the detachment and separation of astrocyte foot processes (ACfps) (Figure 4D,E).

### 2.4. LPS Treatment Induced Activated Brain Endothelial Cells (aBECs)

**Figure 5:**
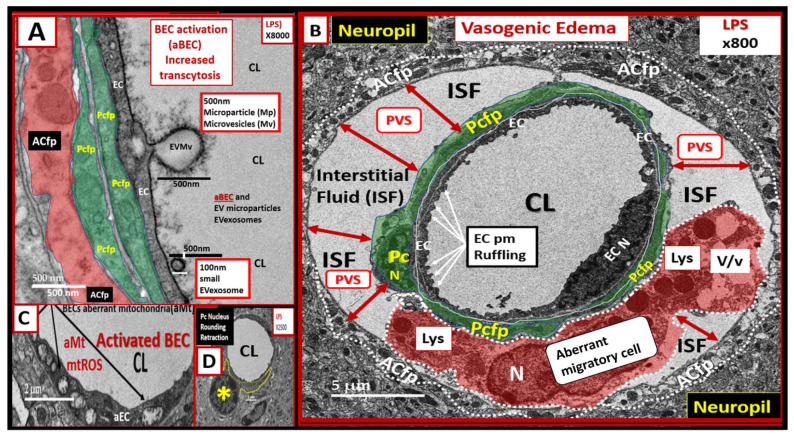
LPS treatment induces activated brain endothelial cells (aBECs). (**A**) depicts an aBEC with the formation of an extracellular vesicle microvesicle (EVMv) and a small EVexosome (EVexo). Pericyte foot process (Pcfp) is shown in pseudo-colored green and astrocyte foot process (ACfp) is shown in pseudo-colored red. (**B**) is the full-sized image of Figure 4D,E and depicts an aBEC with plasma membrane (pm) ruffling (white arrows) that is known to be associated with increased BEC macropinosomes and transcytosis. The aberrant migratory cell (pseudo-colored red) is associated with the expansion of the interstitial fluid (ISF) perivascular space (PVS) (double red arrows). Note that the ACfps are detached and separated from the shared basement membrane of endothelial cells (BECs) and pericytes. (**C**) depicts an aBEC with aberrant mitochondria (aMt) (arrows) with hyperlucency and loss of cristae. (**D**) depicts pericyte nucleus rounding (asterisk) and retraction of Pcfps. The control images can be viewed and compared to these aberrant remodeling changes from Figure 1A, Figure 2A,C, Figure 3A and Figure 4A. Magnification and scale bars vary and are included in each panel. CL = capillary lumen; EC = brain endothelial cells; ISF= interstitial fluid; Lys = lysosome; mtROS = mitochondria reactive oxygen species; N = nucleus; Pc = pericyte; TJ/AJ = tight and adherens junctions; V = vacuole; v = vesicles.

The BECs from the LPS-treated mice demonstrates multiple features of BEC activation (Figure 5).

These features of aBECs include an apparent budding of extracellular vesicles comprised of both larger extracellular microvesicles (approx. 500 nm in size) and smaller exosome-like vesicles (approx. 100 nm) at the luminal membrane (Figure 5A); luminal plasma membrane ruffling (Figure 5B); increased transcytosis with demonstration of both micro- and macropinocytosis (Figure 2 and Figure 3); regions of BEC thickening and hyperlucency; and increased aberrant mitochondria (aMt) (Figure 5C). Figure 5B depicts the single image compatible with vasogenic edema that we have identified to date in this study [22], which also includes an aberrant migratory cell (aMC), most likely a migratory pericyte or leukocyte (a patrolling monocyte and/or a monocyte-derived macrophage, or a dendritic cell). These findings also support the occurrence of diapedesis where the leukocyte is still restricted from entering the parenchyma by the basement membrane [19,20]. Even though there is a complete aMC within this PVS, it is not possible to accurately identify this cell by ultrastructural characteristics alone. However, the presence of multiple lysosomes and vacuoles/vesicles with a paucity of mitochondria suggests that it may be a pericyte that has lifted from the NVU BEC basement membrane and is now possibly migrating within the expanded PVS.

## 3. Discussion

The BBB interface protects the brain microenvironment from the influx of neurotoxic plasma components, aids in ridding it of endogenous toxins, supplies the brain with its nutritional needs, and is part of a brain–body communication axis. All of these functions of the BBB and the communications among the cells of the NVU are adversely affected by systemic inflammation and CNS neuroinflammation. The characteristics of the BBB are determined largely by the communications among BECs and other cells of the NVU, most notably pericytes, astrocytes, and microglia. Here, we examined the ultrastructural effects of neuroinflammation induced by LPS on the morphology of the components of the NVU and the BBB.

Intraperitoneal injections of LPS resulted in numerous ultrastructural remodeling changes to the NVU at a timepoint, and the dosing regimen that we had previously used caused robust BBB disruption to both large (i.e., ^125^I-albumin) and small (i.e., ^14^C-sucrose and ^99m^Tc-DTPA) molecular weight tracers [9,23]. The changes we observed consisted of (*i*) the attraction of MGCs to the immediate vicinity of the NVU; (*ii*) increased BEC transcytosis, which was apparent from the increased numbers of vesicles (coated and non-coated micropinocytic and macropinocytotic vesicles); (*iii*) remodeling changes of Pcs, including rounding of the Pc nucleus and the retraction of pericyte foot processe(s) (Pcfp); and (*iv*) activation of BECs with excretion of extracellular vesicle microvesicles and small extracellular vesicle exosomes, plasma membrane ruffling, and aberrant Mt. Additionally, these remodeling changes were associated with an expansion of the perivascular space that contained an aMC within the immediate vicinity of the remodeled NVU. Furthermore, we speculate that these reactive and atMGCs can result in the polarization of astrocytes to result in detached and separated reactive astrocytes (rACs) from the brain EC [24,25].

Here, it is important to note that we did not measure functional biochemical endpoints of neuroinflammation in this particular mouse cohort because this is an ultrastructural study. It is difficult to perform in tandem functional studies with TEM studies since brain fixation methods are specialized and require perfusion with EM fixatives. Therefore, these fixed tissues are not compatible with biochemical/functional methodologies and are not feasible in the same mouse models. However, the 3 mg/kg of LPS injection regimen used in this experiment has been previously validated by the authors to cause BBB disruption with ^14^C-sucrose, ^99m^Tc-DTPA, ^99m^Tc-albumin, and cytokine/chemokine elevations (including RANTES) in the brain and blood of adult male CD-1 mice [9,23]. Notably, these publications used separate cohorts of mice and were conducted over many years, thus highlighting the reproducibility of our findings that this particular injection regimen of LPS induces BBB disruption and systemic inflammation and importantly neuroinflammation. Therefore, we did not conduct duplicitous experiments in this mouse cohort in this experiment. Furthermore, these previous experiments in the same mouse model, with the same LPS dose (3 mg/kg) and the same injection times, demonstrated significant elevations in ionized calcium-binding adapter molecule 1 (Iba-1), F4/80, and glial fibrillary acid protein (GFAP) that were compatible with neuroinflammation and reactive astrogliosis.

Since LPS does not cross the BBB [17], we assume that LPS initially activates BECs with ensuing changes to the NVU. However, LPS can exert its effects on the NVU by other mechanisms as well, such as an increased passage across the BBB of cytokines and immune cells [8]. Recently, it was shown that the chemokine CCL5 was a mediator of microglial recruitment to the brain vasculature during inflammation [16]. The source of CCL5 in vivo could not be identified in this ultrastructure study; however, prior work has shown that CCL5 secretion by brain endothelial cells and other cell types of the NVU, as well as CCL5 transport across the BBB in the brain-to-blood direction, are increased following LPS treatment [24,26]. The CNS chemokine CCL5/RANTES is, thus, a possible chemotactic mediator of MGCs to the NVU during systemic inflammation [15,16].

Pcs are pluripotent cells and their properties include contractility, antigen-presenting functions, phagocytosis, migration, and capability to undergo transdifferentiation to form adult mesenchymal and other cells [26]. Pcs are the first cells to induce barrier function in the embryonic brain [27]. Pcs synthesize and secrete numerous cytokines, vascular endothelial growth factor (VEGF), platelet-derived growth factor beta receptor (PDGF-βR), basement membrane components, neuron-glial antigen 2 (NG2) proteoglycan, and alpha-smooth muscle actin (αSMA), whereas BECs synthesize and secrete PDGF-β, endothelial nitric oxide synthase (eNOS that is critical for the production of bioavailable nitric oxide), endothelin-1 (ET-1), and von Willebrand factor [21,26,28,29,30,31,32]. The interactions between BECs and Pcs are critical for the proper homeostasis of the NVU [32]. We found that Pcs undergo profound ultrastructural remodeling in the LPS-treated mice, depicting an ultrastructural morphologic phenotype consisting of cellular contraction, rounding of their nuclei, and retraction of the Pc foot process(s) (Pcfps), as well as some surrounding edema (Figure 4). These ultrastructural remodeling changes could be related to the decreased pericyte coverage of the NVU found in the LPS-treated models (Figure 4) [33,34]. Notably, Dore-Duffy et al. have demonstrated that pericytes are capable of detaching and migrating to sites of injury in the CNS, such as those that occur in traumatic brain injury [30]. Presently, it is unclear whether the morphological changes in pericytes that we observed are associated with migration away from the vasculature since we only evaluated a single timepoint. We do note that the identity of the aberrant migratory cell within the expanded perivascular space shown in Figure 5 is unknown; although it may be a leukocyte that has trafficked across the BBB, we cannot rule out that it may also be a migratory pericyte. Notably, these ultrastructural changes observed in the aberrant migratory cell within the PVS are very similar to the changes that Cai et al. have observed with reactive migrating Pcs in stroke injury models [35]. In addition, it should be noted that Owen et al. have demonstrated that once peripherally-derived leukocyte transmigration has occurred, these cells are capable of residing and migrating within the PVS [22]. Therefore, this aMC could possibly be an inflammatory-derived cell; however, we cannot support this with any identifiable morphological and cytological features suggesting an inflammatory cell, such as a roaming patrolling peripheral monocyte-derived macrophage or a reactive microglia morphotype, as described in the diabetic *db*/*db* models or in sepsis-induced neuroinflammation, respectively [3,36,37]. Furthermore, Sirtuin 3 (SIRT3) is important in preserving vascular integrity by targeting pericytes in LPS-treated models [38]. Indeed, Pc structural plasticity plays a critical role in cerebrovascular health.

We did not observe any marked AC or ACfp remodeling except when they become detached and retracted in the images depicting vasogenic edema as shown in Figure 4 and Figure 5. However, we only studied the cortical layer III regions in the frontal region of the brain, and it may be that such changes occur in other regions or at time points which we did not investigate. Others have also observed and described similar remodeling changes in models with increased vascular permeability in neuroinflammatory models [22]. Similar ACfp detachment and separation have been identified in the diabetic *db*/*db* models [3]. The detachment and separation of ACfps with their membraneous AQP-4 could contribute to the observed pericapillary edema. Indeed, the separation of ACfps could not only be a result of the expansion of the perivascular space but could also contribute to pericapillary edema and vasogenic edema [39]. However, we only observed this single instance of vasogenic edema in this experiment. Notably, this pericapillary-perivascular edema suggests that vasogenic edema could contribute to impaired cognition as it has recently been found that enlarged perivascular spaces are negatively associated with normal cognitive assessment [40].

We observed many of the classic characteristics of endothelial cell (EC)–BEC remodeling and activation, including (1) increased transyctotic vesicles; (2) increased plasma membrane (pm) ruffling; (3) increased extracellular microvesicles (EVMv) and extracellular vesicle small exosomes (EVexosomes); (4) increased pinocytosis/transcytosis including micro- and macropinosomes; and (5) increased aberrant mitochondria that are hypolucent with loss of crista (Figure 2, Figure 3 and Figure 5C). Other characteristics of activated or remodeled EC include increased reactive oxygen species (ROS); increased mitochondrial ROS (mtROS), nicotinamide adenine dinucleotide phosphate oxidase (NADPH Ox), and cyclooxygenase-2 (COX-2); increased endoplasmic reticulum stress with a prominence of the Golgi Apparatus; increased attenuation and/or loss of the endothelial glycocalyx; upregulation of endothelial intercellular adhesion molecule 1/vascular cell adhesion molecule 1 (ICAM-1/VCAM-1) receptors; increased leukocyte, platelet, and RBC adhesion; increased BEC stiffening by atomic force microscopy; and EC contraction and shortening with lifting and separation [41,42,43,44]. The increased number of vesicular structures we observed is consistent with prior literature that inflammation induces vesicular/transcytotic pathways of BBB disruption [20], although we did not observe fused caveolae that form transendothelial channels that have been described in other CNS inflammatory/injury models. We posit that, in our LPS model, unfused macro- and micropinocytic vesicles are the predominant mediators of disruption through increased transcytosis of plasma.

Surprisingly, we found no changes in TJ/AJ structures (Figure 2). Such changes are readily observed when LPS is applied to in vitro BBB models using immunofluorescent microscopy techniques [9]. However, in vivo, a relatively high LPS dose is needed to cause widespread BBB disruption [9,23], whereas lower LPS doses can cause changes in transporters in the absence of detectable BBB disruption [45]. Additionally, it has been shown in another neuroinflammatory model of experimental autoimmune encephalomyelitis that tight junctions remain morphologically intact despite neuroinflammation, BBB disruption, and leukocyte trafficking [46]. It may be that regional variations in the extent of LPS-induced disruption could also account for our lack of observation of TJ/AJ ultrastructural changes in this study. We also note that a lack of ultrastructural changes in TJ/AJs as observed by using TEM does not rule out an involvement of paracellular pathways of BBB leakage in this model. It may be that leakage by perivascular routes occurs in the absence of apparent structural changes in TJs/AJs, and TEM with tracers, such as horseradish peroxidase, would be needed to explore this further. It is possible that without the aid of a probe, such as horseradish peroxidase, a paracellular opening of several nm could go undetected.

These ultrastructural observations are consistent with and could underlie the increased permeability [9,10,16,23,47] known to occur with LPS-induced inflammation. Zhu et al. demonstrated by using TEM that an attenuation or loss of the ecGCx was associated with an increase in BEC micropinosomes [48]. Additionally, the loss of VE-cadherin at the adjacent paracellular junctions is related to both clathrin-mediated and caveolae-mediated endocytosis/transcytosis [49]. Our findings are supported by those of Zhang Y et al., in that they found both clathrin- and caveolae-mediated endocytosis was induced by LPS. Clathrin-mediated endocytosis was initially dominant after LPS, but later the dominant pathway was caveolae-mediated endocytosis/transcytosis. Furthermore, Zhang et al. suggested that both decreases in clathrin-mediated and caveolae-mediated endocytosis of VE-cadherin contributed to increased vascular hyperpermeability, since VE-cadherin is involved in the structure of the adherens junction (AJ). They also found that LPS treatment increased tyrosine14 phosphorylation of Cav-1 that also correlated with caveolae-mediated endocytosis of VE-cad, and the development of actin stress fibers that was related to the switch from early clathrin-mediated and later caveolae-mediated endocytosis/transcytosis [49]. Although our results did not identify structural changes in TJ/AJs, it is plausible that such molecular changes could occur in our model and influence paracellular permeability in the absence of overt ultrastructural alterations.

The limitations to this study include the small number of studied mice (n =2 in controls and n = 2 in LPS-treated models); images were taken at a single time point after LPS injections and dynamic aspects were implied; and our tissue were taken from only the frontal cortical layer III regions of the brain. TEM studies are largely morphological in nature and so biochemical and physiological events, consistent with those morphological images, can be inferred based on use of a well-characterized model of BBB disruption, but these were not directly demonstrated in this experiment. However, our findings have elucidated the ultrastructural changes at the BBB that offer new information on the mechanisms of BBB dysfunction following a peripheral inflammatory insult.

To date, the majority of work investigating BBB disruption has focused on disturbances in tight and adherens junction proteins, which regulate paracellular leakage. In contrast, ultrastructural studies of the vesicular/transcellular routes of BBB disruption have been less frequently studied. Our experimental study has filled in some of our gaps in knowledge in regard to increased permeability and neuroinflammation due to LPS administration. For example, and to our current knowledge, our findings are among the first to clearly identify and quantify the different sizes and types of vesicles that appear in BEC capillaries following a sublethal regimen of LPS that has been well-characterized for its effects on BBB functions [9,23,50]. Indeed, these findings highlight a need to further investigate the important contribution of vesicular pathways to aspects of inflammation-induced BBB dysfunction.

## 4. Material and Methods

### 4.1. Animals

All animal studies were performed under the protocols approved by the VA animal care and use committee in accordance with the IACUC guidelines. The studies were conducted in an AAALAC-accredited facility where all mice were maintained on a 12:12 light/dark cycle and received food and water ad libitum. LPS from *Salmonella typhimurium* (Sigma, St. Louis, MO, USA) was dissolved in normal saline and administered to 6–8- week-old male CD-1 mice by giving three intraperitoneal injections at a dose of 3 mg/kg per injection at time (t), including at t = zero, t = six, and t = 24 h, and the mice were sacrificed at t = 28 h post initial injection. All control CD-1 male models received saline (the vehicle for LPS) injections without LPS at the same time intervals and were sacrificed in parallel with the LPS-treated mice. This LPS injection regimen produces robust cytokine responses [50], outward signs of sickness behavior [51], significant weight loss [9,52], and consistently induce BBB disruption to both large and small molecular weight radiotracers [9,23]. All mice receiving repeated LPS injections at this dose survived to the endpoint in the present study.

### 4.2. Tissue Collection and Preparation for Transmission Electron Microscopy (TEM)

The mice that had been anesthetized with urethane were first perfused through the left ventricle of the heart with ice-cold PBS at a rate of 10 mls/min, followed by perfusion with 35 mls standard TEM fixative (2% paraformaldehyde and 2% glutaraldehyde in 100 mM of sodium cacodylate buffer, pH = 7.35) at a rate of 7 mls/min. Their brains were immediately collected and sliced into 1 mm coronal sections, and then placed and stored in a standard TEM fixative for at least 24 h and up to 1 week. These specimens were then rinsed with 100 nM sodium cacodylate buffer (pH 7.35) containing 130 mM sucrose. Secondary fixation was performed using 1% osmium tetroxide (Ted Pella, Inc., Redding, CA, USA) in a cacodylate buffer using a Pelco Biowave (Ted Pella) operated at 100 W for 1 min. The specimens were next incubated at 4 °C for 1 h, then rinsed with a cacodylate buffer, and further rinsed with distilled water. En bloc staining was performed using 1% aqueous uranyl acetate and incubated at 4 °C overnight, then rinsed with distilled water. Using the Pelco Biowave, a graded dehydration series (e.g., 100 W for 40 s) was performed using ethanol, transitioned into acetone, and the dehydrated tissues were then infiltrated with Epon resin (250 W for 3 min) and polymerized at 60 °C overnight. Ultrathin sections were cut to a thickness of 85 nm using an ultramicrotome (Ultracut UCT, Leica Microsystems, Wetzlar, Germany) and stained using the Sato’s triple lead solution stain and 5% aqueous uranyl acetate. Multiple images were acquired for each study group at various magnifications with a JOEL 1400-EX TEM JEOL (JEOL, Peabody, MA, USA) at 80 kV on a Gatan Ultrascan 1000 CCD (Gatan, Inc., Pleasanton, CA, USA).

### 4.3. Ultrastructural and Statistical Analysis

#### 4.3.1. Methods for Determining the Number of Attracted Microglial Cells to the BECs of the NVU

Conventional TEM determination and classification of different cellular morphotypes can be complicated due to an insufficient number of cells in the field of view; therefore, some images were taken at a lower magnification in order to fully evaluate the surrounding tissue as in the hand-counting of attracted MGCs (atMGCs). We studied the models at 800× magnification; scale bar = 5 μm (n = 2) in order to increase our field of view around each NVU. We hand-counted the numbers of atMGCs to the NVUs in 6 different randomly chosen NVU for a total of 12 NVU in each of two mice per group. The atMGCs were defined as being within 20 μm of a capillary NVU. The numbers of atMGCs in each field were then combined and compared using Student’s two-tailed *t*-test in the GraphPad Prism 8 version 9.5.0 software (GraphPad Software, Inc., San Diego, CA, USA).

#### 4.3.2. Methods for Determining of the Number of BBB Vesicles

A high magnifications at 8000× or greater were necessary to obtain a sufficient resolution to evaluate micropinocytic vesicles that ranged from 60 to 70 nanometers (similar in size to caveolae), while macropinocytic vesicles ranged from 125–260 nm in diameter. Because of the marked increase in micro- and macropinocytic vesicles, we decided to randomly examine 6–12 BBB NVU capillary BECs in the control and LPS-treated models (n = 2 mice for the controls and LPS-treated models). The numbers of quantified vesicles in each field were then combined and compared using Student’s two-tailed *t*-test in the GraphPad Prism 8 software version 9.5.0 (GraphPad Software, Inc., San Diego, CA, USA).

## 5. Conclusions

We found that LPS-induced neuroinflammation resulted in profound changes in the ultrastructure of the NVU. This included changes in BECs, astrocytes, pericytes, microglia, and the perivascular space. The main ultrastructural remodeling changes we found in BECs were increases in macro- and micropinocytotic vesicles, plasma membrane cell ruffling, mitochondrial changes, and secretion of extracellular vesicles. We also observed detachment of astrocytes with retraction from BECs and Pc basement membranes; rounding of the pericyte nuclei with a marked decrease in the typically elongated Pc cytoplasmic processes; pericyte retraction from BECs; an increase in the lysosomal, vacuolar, and vesicular compartments of the pericyte; and an increase in the number of attracted ameboid-like microglia near the microvasculature NVUs. We observed no appreciable changes to TJ/AJ. These results support a remodeling ultrastructural morphological basis for the leaky, disrupted BBB known to be present with LPS administration, as well as a dysfunctional NVU.

## Data Availability

Data and materials will be provided upon reasonable request.
